# Account of an Enormous Abdominal Tumour

**Published:** 1846-04

**Authors:** A. B. Longshore

**Affiliations:** Wyoming, Penn.


*Account of an Enormous Abdominal Tumour.* By A. B. Longshore, M. D., of Wyoming, Penn.

To Prof. R. M. Huston.

*Dear Sir*,—In accordance with my promise I will endeavour to give you a brief history of the tumour that I sent you some time since, being compelled, however, to rely for my information mainly upon Drs. Smith and Lewis.

Mrs. B———, æt. 37, from whom the tumour was taken, was the mother of ten children, was of medium size, rather full habit, and sanguine temperament. She usually enjoyed good health, excepting at two different periods, when she threw off false conceptions or moles, accompanied with profuse hemorrhage, and, in her last labour, it became necessary to use instruments, by the aid of which she was delivered of a living child. She soon, however, recovered her accustomed health and strength. She stated that the catamenia returned regularly in six months after the birth of all her children, except the last, and continued so until she again became pregnant. She nursed the last child until it was fifteen months old, and it was sometime after this before the catamenia appeared, and was then accompanied with much pain, and profuse hemorrhage. These paroxysms occurred at irregular intervals, until the formation of the tumour, when they became more regular.

In June, 1838, she first complained of an uneasy sensation in the left iliac region, and in two or three months after, a small tumour could be felt, by pressing with the hand just above the pubis and a little to the left of the linea alba, accompanied with some tenderness—although her general health appeared to be good. On discovering these symptoms she became alarmed and called a consultation of physicians, who pronounced her labouring under ovarian disease,

Dr. Lewis, after having seen the patient several times in consultation with the family physician—Dr. Scott—was called in February, 1839, to attend her. He says:

"I found her in great pain, profuse hemorrhage, and occasional syncope. The tumour had now reached the umbilical region on the left side, and on the right could be felt above the pubis. Upon an examination per vaginam, the os uteri could not be felt, but an elastic irregular mass could be distinguished in the cavity of the pelvis, rather on the right side, exterior to the vagina. During the pains, which much resembled those of labour, the tumour (for I was fully satisfied that it was a tumour, and not a dropsy, as had been represented,) would contract and relax when the pain subsided, which made it evident that it possessed contractile powers, and therefore could not be dropsy. The usual means were resorted to for hemorrhage, viz., the tampon, cold applications, acetate of lead, opium, &c., under which it subsided. From this time forward, until her death, at every menstrual period, the same symptoms, viz., pain, hemorrhage, syncope, &c., would occur, giving way to the same treatment, the tumour gradually increasing."

For the following particulars in regard to this case, I am indebted to Dr. Smith. He says, "that in April, 1843, there was a consultation held as to the propriety of tapping the patient for ascites—a few days previous to this time, the abdomen became so much distended, that (to use her own words) it seemed as though the flesh would be torn from the ribs and breast bone. On the day prior to the assembling of the physicians there was a spontaneous discharge of a quantity of bloody fluid, which had rather a urinous smell. This gave so much relief that the operation was abandoned. Owing to the pressure of the tumour upon the neck of the bladder, there was at times much suppression of urine, which had to be relieved by the use of the catheter, which was often attended with great difficulty, owing to the displacement of the parts. Her appetite was good, and her food appeared to be well digested during the development of the tumour, except at her menstrual periods. Notwithstanding this she gradually became much emaciated, from the constant drain upon the system by these periodical hemorrhages." The day previous to her death I called to see her, in company with Dr. Lewis; she was just recovering from one of the periodical attacks, during which she had discharged an immense quantity of blood, estimated by her attendants at four or five gallons; this, no doubt, was much exaggerated, although the quantity was probably great, as she was left almost completely bloodless—was suffering much from pain, and labouring under great dyspnœa, which had been a prominent symptom for the last two or three years, owing, no doubt, to the pressure of the tumour against the abdominal viscera, pushing them against the diaphragm, and thus compressing the lungs and heart. This, in all probability, was regarded as the main symptom, by those who pronounced her labouring under ascites and hydrothorax, (for she was treated by one or two for these diseases,) looking upon the enlargement of the abdomen as a sign of the former, and the dyspnœa as a sign of the latter. The abdomen was enormously distended by a hard substance, without the least sign of fluctuation, and from its immense size she had been unable to turn herself in bed, for the last two years, without assistance. She died on the 14th of December, 1843.

An autopsy was made twelve hours after death: present, Drs. Smith, Lewis and myself. Placing the body upon the left side, I proceeded to open the abdomen, bv making an incision from the ensiform cartilage to the pubis, along the linea alba. Upon opening the peritoneal sack, six or eight ounces of cream coloured fetid fluid exuded, this was all the fluid found in the cavity. After making the entire section, the abdominal parietes were drawn back, and the whole mass exposed to view, filling the whole abdominal cavity, and starting the cartilages of the ribs outwards, and the ensiform directly forwards. The tumor was attached by strong fibrous bands to the right ramus of the pubis, and right ilium around to the spine; there were also slight adhesions to the descending colon, as far as the sigmoid flexure, and it was attached also to several parts of the abdominal parietes, in front to the bladder, and behind to the uterus and vagina. The attachments were divided, and the vagina cut transversely, leaving the uterus partly imbedded in the mass. Upon examining the cavity of the abdomen, after the removal of the tumour, we found the stomach, liver and intestines, pressed up under the ribs and sternum, encroaching upon the heart and lungs. The viscera presented a healthy appearance, except a preternatural paleness, owing no doubt to the exanguious state of the patient at the time of her dissolution. No examination of the thoracic viscera was made, in consequence of the lateness of the hour, and the distance we were from home. The tumour, which is now in the museum of the Jefferson Medical College, is of a fibrous or fibro-cartilaginous character, with numerous small lobes, attached by pedicles. Some with a broad, and others with a narrow base. The uterus was partly buried in the substance of the tumour, the left ovary, fallopian tube and broad ligaments, were entirely imbedded in it; the right were fully exposed to view, and appeared to be perfectly healthy.

On the front of the tumour there was a distinct raphe, extending from the fundus to the neck, which had its attachment on each side to the neck of the uterus, showing that it originated by two distinct tumours which gradually coalesced as they were developed, until they became firmly united, forming but one mass. We punctured the mass, to the depth of several inches, and laid open several of the lobes, without finding any fluid or cavity; the cut surface was mottled, the external having a pink hue. The tumour weighed fifty pounds immediately after its removal.

The cause or causes of this morbid growth are not known, but in all probability there had been some injury inflicted on the neck of the uterus by the instruments used at her last accouchment. She stated, during her life, that the first time she felt any uneasy sensation in the part, was immediately after bathing her feet in a cold spring when her body was excessively warm, which, very probably, was the immediate or exciting cause.

The most singular circumstances in regard to the case, were the apparent contractile power of the tumour, and the immense quantity of fluid discharged at every menstrual period.

